# MRKNs: Gene, Functions, and Role in Disease and Infection

**DOI:** 10.3389/fonc.2022.862206

**Published:** 2022-04-08

**Authors:** Tongtong Wang, Wenqiang Liu, Changfa Wang, Xuelian Ma, Muhammad Faheem Akhtar, Yubao Li, Liangliang Li

**Affiliations:** ^1^ College of Agronomy, Liaocheng University, Liaocheng, China; ^2^ Veterinary Medicine, Xinjiang Agricultural University, Urumqi, China

**Keywords:** makorin RING finger protein (MKRN), disease, infection, regulation, role

## Abstract

The makorin RING finger protein (MKRN) gene family encodes proteins (makorins) with a characteristic array of zinc-finger motifs present in a wide array from invertebrates to vertebrates. MKRNs (MKRN1, MKRN2, MKRN3, MKRN4) as RING finger E3 ligases that mediate substrate degradation are related with conserved RING finger domains that control multiple cellular components *via* the ubiquitin-proteasome system (UPS), including p53, p21, FADD, PTEN, p65, Nptx1, GLK, and some viral or bacterial proteins. MKRNs also served as diverse roles in disease, like MKRN1 in transcription regulation, metabolic disorders, and tumors; MKRN2 in testis physiology, neurogenesis, apoptosis, and mutation of MKRN2 regulation signals transduction, inflammatory responses, melanoma, and neuroblastoma; MKRN3 in central precocious puberty (CPP) therapy; and MKRN4 firstly reported as a novel E3 ligase instead of a pseudogene to contribute to systemic lupus erythematosus (SLE). Here, we systematically review advances in the gene’s expression, function, and role of MKRNs orthologs in disease and pathogens infection. Further, MKRNs can be considered targets for the host’s innate intracellular antiviral defenses and disease therapy.

## Introduction

Orthologs of the Makorin RING finger protein (MKRN) have been found in fungi, plants, and mammals (1). The *mkrn* gene family encodes proteins with unique zinc finger arrays, including C3H motifs, a new Cys-His motif, and a RING finger. The ancestral founder of this gene family is *mkrn1*. Another component, *mkrn2*, may have evolved 450 million years ago due to *mkrn1* gene duplication (2). *mkrn3* is an intronless gene found on chromosome 15’s long arm in the Prader-Willi syndrome (PWS) crucial area (3). *mkrn4* has been previously known as MKRNB (4).

Ubiquitination is a post-translational modification mechanism involved in several biological processes, including cell survival, differentiation, innate and adaptive immunity. Ub is covalently coupled to the target protein with single or multiple 76 amino acid globular protein by activating (E1), conjugating (E2), and ligating (E3) enzymes (5). These three types of enzymes work in a certain order. E1 activates Ub before it is passed to an E2 conjugating enzyme. Following that, E3 ubiquitin ligases attach to E2 and the substrate, principally supplying the Ub chain to the substrate and promoting isopeptide synthesis. Finally, the 26S proteasome degrades the target protein into tiny peptide fragments (6, 7). Indeed, the presence of the RING finger domain is the most noticeable structural feature of MKRNs, not only because this region is found in proteins that form repressive complexes to reduce target gene activity (8), but also because it is a signature domain of E3 ubiquitin ligases (9). This review will concentrate on the function of E3 ubiquitin ligases to mediate substrate degradation by UPS of MKRN1, MKRN2, MKRN3, and MKRN4. Special attention is also focused on the potential role of MKRNs in physiological functions and disease regulation.

## *mkrn* Gene and Expression

Makorin RING finger protein (MKRN) is produced by the *mkrn* gene family, distinguished by the intron-containing founder of the intronless and a high level of sequence conservation in taxa spanning from invertebrates to vertebrates. Nine *mkrn* family loci spread throughout the human genome (2). So far, four functional *mkrn* genes *mkrn1*, *mkrn2*, *mkrn3* and *mkrn4* have been elaborated in literature. *mkrn1* is ancestral gene of this family and characterized in humans, mice, wallabies, chickens, pigs drosophila, nematode and plants (10, 11). MKRN1 is substantially and ubiquitously expressed in human organs, including the hypothalamus and the amygdala, according to expression assessments. When transfected into different cell types, MKRN1 is expressed in the nucleus and cytoplasm.

The identification and characterization of the *mkrn2* locus in yellowtail fish aided research into the makorin gene family. *mkrn2* orthologs are found in humans, mice, and zebrafish. *mkrn2* is assumed to have evolved from an ancestral *mkrn1* by gene duplication 450 million years ago, and it (*mkrn2*) partially overlaps with the *raf1* protooncogene in an antisense transcriptional direction. MKRN2 is found in all human tissues and cell lines, according to expression studies (12). MKRN2 expression was greater in primary leukemia samples than in age-matched normal BM cells. However, no significant association was found between MKRN2 expression levels in any leukemia subtypes (13).

*mkrn*3 is specific to therian mammals, and it is an intronless retrocopy of *mkrn1* produced by reverse transcription of an *mkrn1* mRNA molecule. The reverse transcriptase encoded by autonomous retrotransposable elements catalyzes the production of such retrogenes. Several more *mkrn1* retrocopies have been found in mammalian genomes, with the majority of these most likely belonging to pseudogenes (14). The presence of *mkrn3* in the dog, mouse, and human genomes, together with its lack in the chicken, fish, and platypus genomes, suggests that Prader-Willi syndrome (PWS) acquired *mkrn3* critical area about 80-90 million years before (15). MKRN3 is ubiquitously expressed in adult tissues in both mice and humans, with the greatest level in the testis. MKRN3 was found in high concentrations in the brain and lung of human embryonic tissues. *mkrn3* gene expression has been identified in mice from the blastocyst stage and embryonic days 8 to 17 and in ESCs (3).

However, *mkrn4*, a novel member of the makorin gene family, was discovered in poeciliidae fish. MKRN4 has been discovered as having gonad-specific expression in vertebrates (4). MKRN4 shares 81% of its amino acid identity with MKRN1, 46% with MKRN2, and 52% with MKRN3 (16). *mkrn*4 gene has been previously known as MKRNB, although it is also present in the human genome, labelled as a pseudogene (4). MKRN4 expression investigation in medaka, zebrafish (ray-finned fishes), and amphibians revealed a substantially gonad-biased expression pattern, as did MKRN1 and MKRN2, with particularly strong expression in the ovaries (2, 17).

**Figure 1:**
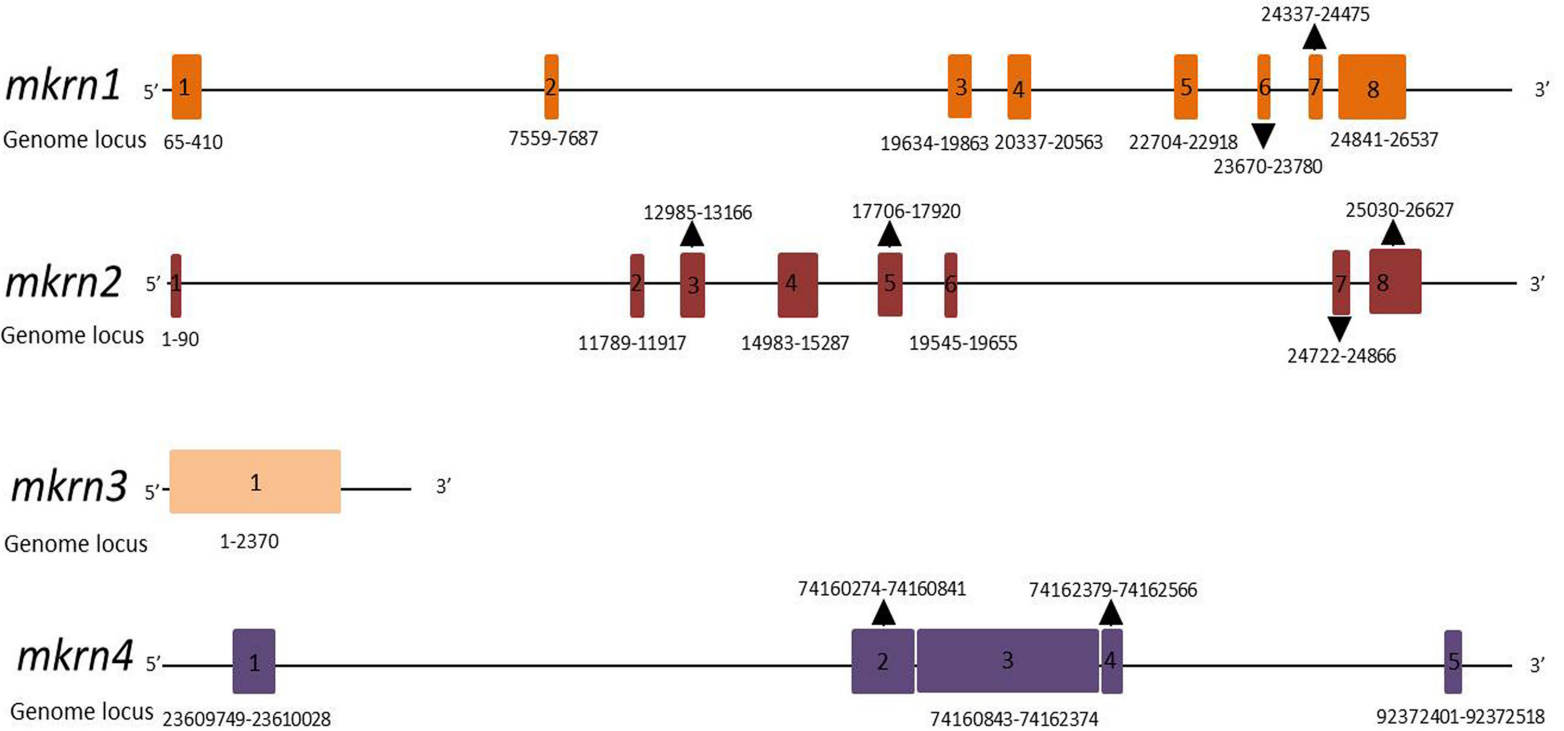
Schematic gene structures of human *mkrn*1, *mkrn*2, *mkrn*3 and *mkrn*4 loci. Exons are represented as boxes. Number and locus of exons in genome are indicated.

The gene structures of the members of the makorin protein family (human *mkrn1*, *mkrn2*, *mkrn3*, *mkrn4*) were analyzed in **Figure 1** to show their similarities and differences. *mkrn1* and *mkrn2* have the same exons, but *mkrn3* has only one exon, *mkrn4* has five exons. All of them possessed different exons about location and sizes.

## MKRN Functional Domains

**Figure 2:**
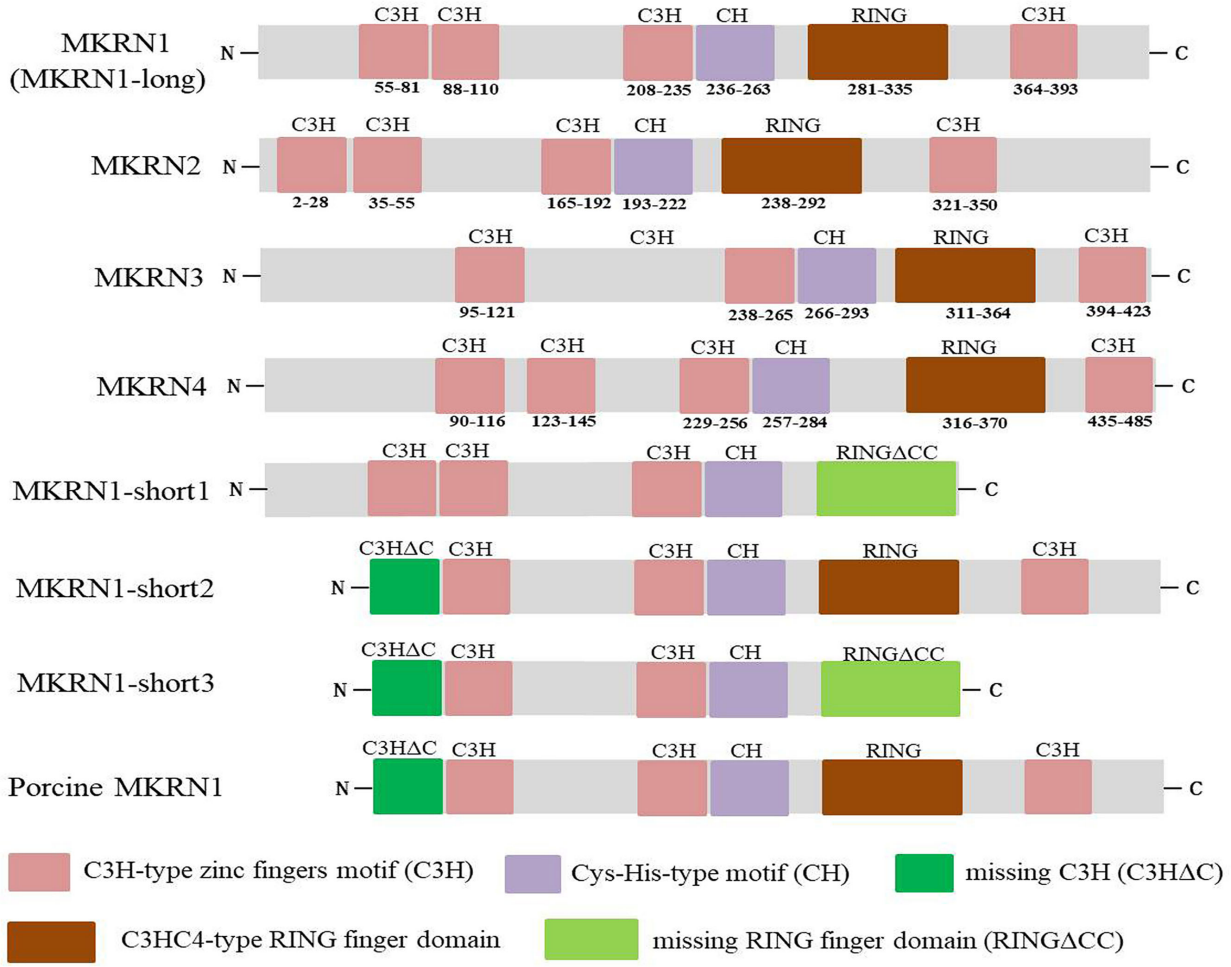
Functional domains of human MKRN1 (MKRN1-long), MKRN2, MKRN3 and MKRN4, the transcript variants of human MKRN1 (MKRN1-short1, MKRN1-short2, MKRN1-short3) and porcine MKRN1. The makorins contain several C3H zinc finger domains (pink), a Cys-His (CH) motif (purple), a C3HC4 RING domain (red), missing C3H motif (C3HΔC) (green) and RING finger domain (RINGΔCC) (aqua).

The *mkrn* gene family encodes different proteins with diverse zinc-finger motif composition and structure, including numerous C3H motifs, a RING finger motif, and a Cys-His motif (2, 3). C3H zinc fingers, which are present in various ribonucleoproteins and may serve as RNA-binding proteins, affect post-transcriptional RNA processing at numerous levels, including alternative splicing, mRNA stability, mRNA localization, and translation efficiency (18, 19). Most E3 ubiquitin ligases have the RING finger domain, which mediates the transfer of ubiquitin from an E2 ubiquitin-conjugating enzyme to target protein substrates (20). **Figure 2** demonstrated the functional domains of human MKRN1, MKRN2, MKRN3, and MKRN4. It showed that MKRN1, MKRN2, and MKRN4 have the same number of C3H-type zinc fingers, but MKRN3 has one less. They all share the identical Cys-His (CH) and C3HC4-type RING finger domains as makorins (**Figure 2**). Human MKRN1 has four isoforms that encoded by a single *mkrn1* gene and emerge *via* alternative splicing and variable polyadenylation. MKRN1-long has four C3H-type zinc fingers, a Cys-His-type motif, and a highly conserved C3HC4-type RING finger domain. MKRN1-short1/MKRN1-short3 human MKRN1 transcript variants are missing the C-terminal ZF and the final 6 amino acids (aa) of the RING finger domain (RFCC), which are required for binding the second zinc ion, or the N-terminal segment (64 aa). Previous research found that the pattern of pig MKRN1 expression is similar to that of human MKRN1-short 2, which lacks the N-terminal 64 aa seen in MKNR1-long (11) (**Figure 2**). Three C3H zinc fingers flank the RING finger domain on its N-terminal side and one C3H zinc finger on its C-terminal side in most plants and invertebrates (but not in drosophila). The cysteine and histidine residues motif (the Cys-His motif) is located between the third C3H zinc finger and the RING domain. These domains are associated with their function, as discussed below.

## MKRN1 Function

### Transcription Regulation

MKRN1 can regulate RNA polymerase II-dependent transcription. As a transcriptional factor, it inhibits not just c-Jun/AP-1 transcriptional activity but also numerous other RNA polymerases II-dependent transcriptional activators, including p53, NF-κB (p65), and the human androgen receptor (hAR) (21), its transrepression action is unrelated to its ubiquitin ligase activity (22). MKRN1 has been reported to act downstream of OCT 4, a transcriptional factor, suggested playing a role in establishing and maintaining totipotency or pluripotency of embryonic and undifferentiated stem cells, embryonal carcinoma cells, and embryonic germ cells *in vitro* (23). The pseudogene trans-regulation model is predicated on active, imprinted transcription of *mkrn1-p1* to stabilize *mkrn1* mRNAs in trans and down-regulate *mkrn1* transcripts to exhibit the reported kidney and bone phenotypes (24). According to this scenario, the mechanism and the pseudogene should be maintained between species since their absence would destabilize MRKN1 and significantly lower reproductive fitness due to severe newborn mortality associated with its disruption. However, *mkrn1-p1* is solely found in mice and not in any other species, including rats. To address this discrepancy, it was postulated that other mammalian species employ distinct *mkrn1*-derived pseudogenes to conduct the identical trans-stabilization of mRNA generated by the *mkrn1* source gene (25).

MKRN1 was also shown to be connected with several RNA-binding proteins, indicating that it is a component of the ribonucleoprotein complex. Because MKRN1 has four C3H zinc finger domains associated with RNA-binding capability, UV crosslinking and immunoprecipitation can be used to examine MKRN1’s ability to interact directly with RNA (CLIP) (20, 26). MKRN1 has a hitherto unknown RNA-binding function, according to this study.

### MKRN1 as an E3 Ubiquitin Ligase

Ubiquitylation is well recognized for directing proteins for destruction by the 26S proteasome, as well as internalization and lysosomal targeting, transcriptional control, protein interaction modulation, subcellular distribution change, DNA repair, and transmembrane signaling propagation (27–30). Ubiquitylation has been connected to almost every biological activity, which is not unexpected. The great majority of E3 ligases are RING-domain E3 ligases (RING) and RING-related E3s, which include plant homeodomain (PHD), leukemia-associated protein (LAP) finger proteins, and U-box family members (9, 31–33). More than 600 possible RING finger domain E3s are encoded in the mammalian genome (34, 35). A classical RING finger is a Zn^2+^-coordinating domain made up of a succession of precisely spaced cysteine and histidine residues that facilitate E2-dependent ubiquitylation (36, 37).

In the search for regulators of the ubiquitination and proteasome-dependent degradation of human telomerase reverse transcriptase (hTERT), *mkrn1* was discovered as a new RING finger gene expressing E3 ligase (38). MKRN1’s E3 ligase activity, on the other hand, is linked to its gene structure in distinct orthologs. MKRN1 has recently been demonstrated to promote the degradation of several substrates *via* the ubiquitin-proteasome system (UPS), including host proteins p53, p21, FADD, PTEN, AMPK1 and 2, as well as viral proteins or bacteria associated with intact RING finger domains (11, 39–43).

MKRN1 interacts with viral proteins and polyubiquitinates these proteins. MKRN1 preferentially targets PCV2 Cap lysine residues and promotes polyubiquitination mediated destruction. Mutation of either of the three lysine residues in the Cap protein or histidine at residue 243 within MKRN1’s RING finger domain abolished MKRN1’s E3 ligase function, making cells incapable of triggering Cap ubiquitination and destruction (11). In a proteasome-dependent manner, MKRN1 may also cause WNVCp ubiquitination and destruction. Interestingly, MKRN1 degraded the WNV Cp mutant with amino acids 1 to 105 deleted, but not the mutant with amino acids 1 to 90 deleted. When three lysine sites at positions 101, 103, and 104 of WNV Cp were replaced with alanine, MKRN1-mediated ubiquitination and mutant degradation were significantly inhibited, indicating that these sites are required for ubiquitination (40).

Recombinant MKRN1 ubiquitinates entire *M. tuberculosis in vitro*, indicating a new potential role for MKRN1 against *mycobacteria* (44), suggesting MKRN1 E3 ligase acts as a defensive effect during pathogens infection.

In HAdV-C5-infected cells, however, the cellular E3 ubiquitin ligase MKRN1 is a unique precursor pVII interacting protein. Surprisingly, the endogenous MKRN1 protein was degraded by proteasomes during the late phase of HAdV-C5 infection in various human cell lines (45), implying that HAdV may have evolved a mechanism to avoid MKRN1-mediated host defensive strategies to benefit their replication. MKRN1 was identified as a possible common target throughout several viral infections.

### Regulation of MKRN1 in Disease

MKRN1 is an E3 ubiquitin ligase that regulates metabolic diseases and malignancies through the ubiquitination of substrate proteins (39, 46). AMPK is implicated in a variety of metabolic disorders, including obesity, type 2 diabetes, fatty liver syndrome, cardiovascular disease, and cancer. AMPK is an appealing target for controlling or curing metabolic illnesses due to its impact on creating brown and beige adipose tissues and mitochondrial regeneration. MKRN1 regulates AMPK/AMPK ubiquitination and proteasome-dependent degradation to maintain its protein homeostasis might have major systemic metabolic consequences, allowing researchers to create innovative treatment techniques that target not just AMPK but also its regulators. An oncogene initiates the senescence process by activating p14ARF and then p53, preventing cells from becoming tumorigenic in the tumor suppression process (39). The endogenic p53 protein is tightly regulated by ubiquitin proteasome degradation pathway induced by negative regulator murine double minute 2 (MDM2), which inhibits the oncogenic action of MDM2 and enhances p53-dependent transactivation and apoptosis (47). However, the tumor suppressor protein p53 is also a transcriptional activator of PTEN, and this inhibits the downstream signaling of phosphatidylinositide 3 (PI3)-kinase, leading to the inactivation of AKT and, eventually, mTOR. In this mechanism, MKRN1 may cause the degradation of p14ARF, p53, and PTEN (39).

The adenomatous polyposis coli (APC) protein, which acts as a negative regulator of the Wnt signaling pathway, is also a tumor suppressor. MKRN1, an E3 ligase, has been shown to bind with and ubiquitylate APC, increasing its proteasome degradation and favorably regulating Wnt/-catenin-mediated biological activities (46). MKRN1 promotes Fas-associated protein with death domain (FADD) substrate ubiquitination and proteasome pathway degradation to delay the cell death-receptor apoptosis cascade activation by caspase 8, lower cell sensitivity to death ligands, and eventually protect against cell death. FADD content rises in MKRN1-depleted cervical cancer cells, indicating increased susceptibility to exogenous apoptotic ligands (41). Surprisingly, MKRN1 expression is reduced in cardiac tissues during intermittent hypoxia (IH). Furthermore, MKRN1 stimulates p21 ubiquitination and proteasome pathway degradation to down-regulate p21 expression, decreasing IH-induced ROS generation and myocardial apoptosis, providing a novel target for lowering cardiovascular risk in obstructive sleep apnea (OSA) patients (48).

In promoting atherosclerosis, MKRN1 expression was uniquely inhibited and contributed to endothelial cell (EC) activation and senescence, in which process TERF2IP S205 was phosphorylated and induced a downstream event of p90RSK activation (49). *mkrn1* gene functions in the metabolic regulation of oogenesis through up-regulating the MKRN1 protein. It functions as a tissue-specific regulator of the insulin/Tor signaling pathway (upstream of Akt/S6K) to stimulate oogenesis in the ovaries in a nutrient-dependent manner (50). In pancreatic malignancies, lncRNA-CF129145.1 (CF129) stimulates the interaction of p53 with the E3 ligase MKRN1, resulting in the ubiquitination and degradation of the p53 protein, which inhibits the proliferation and metastasis of PC cells.

## MKRN2 Function

MKRN2’s possible physiological activities have recently been identified. MKRN2 knockout mice, for example, have been shown to have problems in male fertility as well as abnormalities in testis function (51). Furthermore, MKRN2 has been implicated as a negative regulator of a variety of cellular and physiological pathways, including neurogenesis in *Xenopus laevis* (52, 53), NF-kB signaling in human cells (54), and non-small-cell lung cancer cell metastasis *via* the PI3K/Akt signaling pathway (55). MKRN2 inhibits the p53 apoptosis effector related to PMP22 (PERP) expression, and levels of the protein in sperm samples have an inverse correlation with infertility levels, implying that MKRN2 is important for protecting germ cells from excessive apoptosis and implicating MKRN2-based suppression of the p53/PERP signaling pathway in spermatogenesis and male fertility (56). In addition, specific gene missense mutation of MKRN2 is associated with degenerative lumbar spinal stenosis (DLSS), the major variant type was single nucleotide polymorphism (SNP), and C > T was the most common single nucleotide change (57), and genetic differences in microRNA 154-binding sites, as well as MKRN2, reduce or abolish microRNA-mediated regulation of genes related with cardiometabolic abnormalities (58).

MKRN2 also has E3 ligase activity associated with the RING finger domain, which is bound to p65 and promotes polyubiquitination, proteasome-dependent degradation of p65 *via* the MKRN2 RING finger domain, which inhibits p65-mediated NF-κB transactivation and inflammatory responses (54). Furthermore, MKRN2 controlled melanoma cell proliferation *via* interacting with and ubiquitylating p53, implying that MKRN2 might be a therapeutic target for melanoma (59). Previous research found that IGF2BP3 is a novel ubiquitylating substrate for MKRN2 and that MKRN2 reduces central nervous system tumors by regulating CD44 and PDPN in an IGF2BP3-dependent way. As a result, MKRN2 may be a promising therapeutic target for neuroblastoma (60).

## MKRN3 Function

The RING finger domain of MKRN3 is thought to be a potential E3 ubiquitin ligase that suppresses Nptx1 expression by polyubiquitination before puberty. Genetic variations in and near the MKRN3 gene have been linked to instances of familial and non-familial central precocious puberty (61–64). As a result, MKRN3 has been found to inhibit trigger protein maturation in juvenile puberty delay. The genetic treatment for accurate MKRN3 compensation may be a promising strategy for central precocious puberty (CPP) therapy.

## MKRN4 Function

Because of the lack of functional promoters, integrated, processed pseudogenes have been assumed to be untranscribed and utterly non-functional since their discovery (65). Processed pseudogenes may occasionally gain promoter activity and be transcribed (66, 67). Interestingly, a recent study reported that the novel E3 ligase MKRN4 was identified that induces GLK protein degradation, which suppresses GLK protein overexpression and has a positive SLE function. For the first time, MKRN4 is shown as an E3 ubiquitin ligase instead of a pseudogene (16).

## Conclusion and Future Perspectives

**Figure 3:**
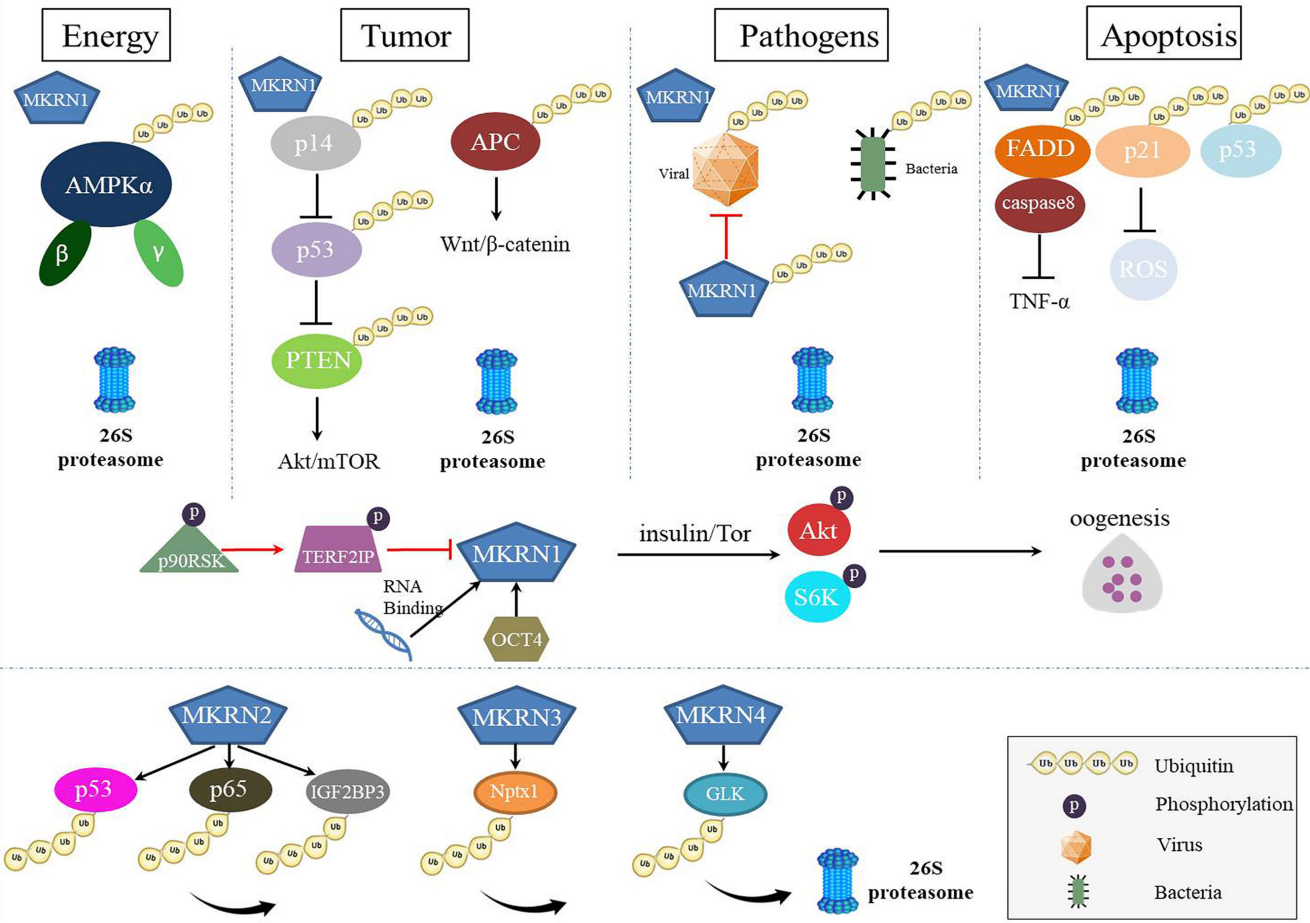
The roles of MKRNs in cells. MKRNs (MKRN1, MKRN2, MKRN3, MKRN4) served as E3 ubiquitin ligase play a leading role in degrading many substrates and regulating several diseases. MKRN1 regulates energy metabolism, tumor suppression, viral protein or bacteria ubiquitination, and apoptosis by degrading their substrates. MKRN1 has RNA-binding functions, up-expressed to regulate the insulin signaling pathway during oogenesis and down-expressed to regulate the senescence-associated secretory phenotype (SASP). MKRN2, MKRN3, and MKRN4 also mediated substrates ubiquitination and degradation.

*mkrn* gene family has similar the RING finger domain required for E3 ubiquitin ligases of the RING finger class, despite a different array of zinc-finger motifs. However, as multiply function proteins, MKRNs served as E3 ubiquitin ligase play a leading role in degrading many substrates and regulating several diseases (**Figure 3**). Thus, in the future, novel therapeutic techniques that target protein-protein interactions and interfere with the binding of MKRNs and their substrates may be developed, and these tactics may create fresh therapeutic options for the treatment of illnesses or pathogens infection.

## Author Contributions

TW and LL conceived and wrote the manuscript. WL, CW, MA, and YL revised the manuscript. All authors contributed to the article and approved the submitted version.

## Funding

This work was supported by grants from the National Natural Science Foundation of China (32002248) and the Natural Science Foundation of Shandong Province (ZR2020QC016, ZR2020QC017).

## Conflict of Interest

The authors declare that the research was conducted in the absence of any commercial or financial relationships that could be construed as a potential conflict of interest.

## Publisher’s Note

All claims expressed in this article are solely those of the authors and do not necessarily represent those of their affiliated organizations, or those of the publisher, the editors and the reviewers. Any product that may be evaluated in this article, or claim that may be made by its manufacturer, is not guaranteed or endorsed by the publisher.
